# National Strategies for Preventing and Managing Non-communicable Diseases in Selected Countries

**DOI:** 10.3389/fpubh.2022.838051

**Published:** 2022-02-10

**Authors:** Lucia Gassner, Ingrid Zechmeister-Koss, Inanna Reinsperger

**Affiliations:** ^1^Austrian Institute for Health Technology Assessment GmbH, Vienna, Austria; ^2^Department of Sport Science, University of Vienna, Vienna, Austria

**Keywords:** non-communicable diseases, chronic illness, national strategies, prevention, disease management

## Abstract

Non-communicable diseases (NCDs) are the leading cause of death worldwide and are responsible for a high burden of disease. Many countries have developed national strategies for the management and prevention of NCDs to improve the care of chronically ill people or prevent NCDs. This article aims to provide an overview of national NCD strategies from selected countries and their implementation. The focus was on cardiovascular and chronic respiratory diseases, diabetes type II, and depression. A comprehensive, structured hand search was conducted in various databases and websites for national strategies on the 4 NCDs. According to pre-defined criteria, 18 strategies from 8 countries (Germany, Switzerland, Netherlands, Finland, Ireland, United Kingdom, Canada, Australia) were selected. The included NCD strategies differ considerably in terms of level of detail, structure and implementation. All strategies include information on planned activities, but only a few provide detailed information on these interventions, including their evaluation. A structured approach from the macro to the micro level seems crucial for a comprehensive, coordinated overall policy. Strategies should be evaluated regularly using appropriate methods to measure target achievement. For the prevention and management of NCDs, it is important to start in early childhood and to adequately consider the social determinants of health with a “Health in All Policies” approach.

## Introduction

Non-communicable diseases (NCDs), also known as chronic diseases, are a globally increasing concern for national governments and society due to their high mortality and morbidity (1, 2). These medical conditions are associated with slow progress and long durations (2). According to the World Health Organization (WHO), they are the leading cause of deaths worldwide (2), accounting for 86% of mortality and 77% of disease burden in WHO European Regions (1). By 2030, NCDs' total number of deaths will rise to 55 million, according to the WHO's projection, if nothing changes. Cardiovascular diseases (CVDs), cancers, chronic respiratory diseases, and diabetes are the top 4 causes of premature mortality among NCDs (2). Nevertheless, people live longer with disabilities resulting from chronic diseases, as premature mortality reduces. Multimorbidity affects 65% of people over 65 years, requiring more patient-centered and complex care models (3).

NCDs are also responsible for growing health inequalities observed in many countries, demonstrating socio-economic gradient and gender differences; e.g., European women live approximately 8 years longer than men (4). NCDs result from several factors, e.g., behavioral, environmental, genetic, and physiological factors (2). They share 4 main behavioral risk factors: unhealthy diet, tobacco, harmful use of alcohol, and physical inactivity (4). Apart from the behavioral health determinants, living conditions, such as poverty or housing conditions, play an essential role in developing NCDs (5). Education is a key factor influencing health because it is related to health literacy and consequently to health behavior and influences labor market opportunities, income (poverty), and other living conditions (5).

Cost-effective curative and preventive action can significantly reduce NCD burden, along with already available NCD control and preventive interventions (4). However, NCDs and their challenges cannot be dealt within the health sector alone. New health policies are required to develop national integrated strategies for NCD control and prevention (6). Globally, a number of documents exist concerning NCD prevention and management on a strategic level. Key documents are the *WHO Global Action Plan for the Prevention and Control of NCDs* (4) and the *Action Plan for the Prevention and Control of NCDs in the WHO European Region 2016–2025* (5). Additionally, individual countries develop multisectoral, comprehensive NCD control and prevention policies. A systematic policy analysis by Briggs et al. evaluated health policies for integrated prevention and management of NCDs among the Member States of the OECD (Organization for Economic Co-operation and Development), focusing on their aims and strategies to achieve these aims as well as evaluating the integration of musculoskeletal conditions. The analysis including 44 policies from 30 OECD Member States found that the policies of most countries covered cancer (83%), diabetes/endocrine disorders (77%), CVDs (77%), mental health conditions (63%), respiratory conditions (63%) and musculoskeletal health and pain (50%). Almost all policies (96%) outlined general strategies (7).

Based on the analysis of Briggs et al., we are extending the analysis by not only including strategies concerning NCDs in general but also considering strategies focussing on single NCDs. This paper, therefore, aims to provide an overview of the latest status of national strategies for preventing and managing selected NCDs (CVDs, chronic respiratory diseases, diabetes type II, depression) in selected (Western high-income) countries and their implementation.

We will further address knowledge gaps concerning the implementation of the strategies and the specific programs and interventions planned to be carried out in order to reach the respective aims of the strategy.

The following research questions will be answered: (i) Which *national strategies/policies* (at a macro level) are in place in Western high-income countries concerning the aforementioned NCDs and their risk factors, and what are their main objectives and features? (ii) What information is available on the *implementation* stage and implementation process of those strategies?

## Methods

### Literature Search of National Strategies

In April/May 2021, we conducted an extensive structured hand search for national strategies and policies addressing the 4 NCDs mentioned above by using the following online resources and databases: websites of national ministries of health, websites of national public health institutions, Google (Scholar), PubMed, WHO website, and OECD website. Additionally, we used the article by Briggs et al. (7) that analyzed integrated prevention and management policies for NCDs among OECD countries as a starting point for our search. We used keywords such as non-communicable diseases, chronic diseases, cardiovascular diseases, chronic respiratory/pulmonary diseases, diabetes, depression, mental health and national strategy or policy. If necessary, we contacted experts from the respective countries for further information and clarification.

### Selection of Countries and National Strategies

The inclusion criteria for relevant national strategies are listed in (Table 1). Together with the Austrian Ministry of Social Affairs, Health, Care and Consumer Protection, who commissioned the corresponding report (8), the pre-defined focus was on CVDs, chronic respiratory diseases, diabetes type II and depression.

**Table 1:** Inclusion criteria for relevant national strategies.

**Description**	**Project scope**
Type of publication	National strategies and policies, which address one of the 4 non-communicable diseases (cardiovascular diseases, chronic pulmonary/respiratory diseases, diabetes type II, depression)
Content	▪ main characteristics and/or ▪ information on the (planned) implementation process
Settings	Western high-income countries (Europe, North America, Australia, New Zealand)
Languages	English, German

We selected a range of countries that provided the most detailed national strategies for one (or more) of the 4 NCDs in English or German. Starting with the article by Briggs et al. (7), giving a good overview of integrated NCD prevention and management strategies among OECD countries, we aimed to include countries with different public health traditions and health systems, focusing on Europe but also involving non-European high-income countries. Additionally, we selected the most recent and the most detailed national strategies addressing one (or more) of the 4 NCDs available in English or German.

### Data Extraction and Analysis of National Strategies

We prepared data extraction tables (available on request) for each of the selected countries, deciding inductively which information to extract. For the extracted interventions of the strategies, we concentrated on adults (rather than children and adolescents), as this focus was pre-defined by the Austrian Ministry. The data extraction tables include *main characteristics*: e.g., title of the strategy, year of publication, indications, focus and aims (research question 1); and information on the (planned) *implementation process*: e.g., time frame, involved stakeholders, organizational framework conditions, evaluation/monitoring, planned activities to reach the strategy's aims (research question 2). The information was summarized across all countries and their respective strategies for each extracted category. A qualitative content analysis was done to identify themes regarding visions and goals of the strategies, as well as the activities and measures. These were clustered into broader categories and narratively described.

## Results

### Included National Strategies

For the analysis of national NCD strategies, we included a total of 18 relevant documents (9–26) from 8 countries. These included 6 European countries (Germany, Switzerland, Netherlands, Finland, United Kingdom, Ireland) as well as Canada and Australia. Table 2 lists all included countries and their strategies.

**Table 2:** Included countries and strategies (*n* = 18).

**Country**	**Title of strategy**	**[ref]**
Germany	▪ IN FORM National Action Plan for the Prevention of Malnutrition, Physical Inactivity, Obesity and Related Diseases [IN FORM Nationaler Aktionsplan zur Prävention von Fehlernährung, Bewegungsmangel, Übergewicht und damit zusammenhängenden Krankheiten]	(21)
	▪ Health Targets [Gesundheitsziele.de]	(9)
Switzerland	▪ National Strategy Prevention of Noncommunicable Diseases 2017–2024 [Nationale Strategie Prävention nichtübertragbarer Krankheiten 2017–2024]	(18)
	▪ National Strategy Cardiovascular Diseases, Stroke and Diabetes 2017–2024 [Nationale Strategie Herz- und Gefäßkrankheiten, Hirnschlag und Diabetes 2017–2024]	(15)
Netherlands	▪ The National Prevention Programme 2014–2016	(22)
Finland	▪ National Mental Health Strategy and Programme for Suicide Prevention 2020–2030	(10)
UK	▪ No Health Without Mental Health: A Cross-Government Mental Health Outcomes Strategy for People of All Ages	(25)
	▪ An Outcomes Strategy for COPD and Asthma	(24)
	▪ Cardiovascular Disease Outcomes Strategy. Improving outcomes for people with or at risk of cardiovascular disease	(23)
	▪ A Diabetes Strategic Framework	(17)
Ireland	▪ National Framework for the Integrated Prevention and Management of Chronic Disease in Ireland 2020–2025	(11)
	▪ Changing Cardiovascular Health. National Cardiovascular Health Policy	(26)
Canada	▪ Improving health outcomes: A paradigm shift. Center for Chronic Disease Prevention – Strategic Plan 2016–2019	(19)
	▪ Changing directions, changing lives: The Mental Health Strategy for Canada	(16)
Australia	▪ National Strategic Framework for Chronic Conditions	(13)
	▪ Australian National Diabetes Strategy	(20)
	▪ National Strategic Action Plan for Lung Conditions	(12)
	▪ The Fifth National Mental Health and Suicide Prevention Plan	(14)

### Main Characteristics

#### Country, Year

We identified 4 relevant strategies from Australia (12–14, 20), 4 from the United Kingdom (17, 23–25), 2 from Germany (9, 21), 2 from Switzerland (15, 18), 2 from Ireland (11, 26), 2 from Canada (16, 19), 1 from Finland (10) and 1 from the Netherlands (22). The strategies were published between 2011 and 2020.^1^

#### Publisher

12 of the 18 strategies were published by the respective ministries of health (10, 12–14, 17, 18, 20, 21, 23–26). 1 strategy was issued by the government (22). Another 3 strategies (11, 16, 19) were developed and published by different organizations at the federal level, such as the Public Health Agency of Canada or the Irish Health Service Executive. 1 strategy was launched by several medical associations (15), and 1 strategy has been developed by a long-term cooperation network of 120 organizations (9).

#### Indications (NCDs)

7 of the included documents address prevention and/or management of NCDs or chronic diseases in general or mention several of the relevant NCDs (9, 11, 13, 18, 19, 21, 22). 11 strategies are directed specifically to one of the NCDs: We identified 4 documents for mental health (10, 14, 16, 25), 2 for chronic respiratory diseases (12, 24), 2 for diabetes (17, 20), 2 for CVDs (23, 26), and 1 strategy that addresses CVDs as well as diabetes (15).

#### Focus: Prevention/Management

The majority of the identified strategies (14) include information on prevention *and* management related to the NCDs (9–17, 20, 23–26). 4 of the documents focus on prevention only (18, 19, 21, 22).

#### Targets/Aims/Visions

Most of the included strategies formulate an overarching aim or vision as well as more specific targets or objectives, which differ, however, between the documents. “Living healthier lives,” “stay healthy,” or “promote individual health” are visions shared by several strategies on NCDs or chronic conditions in *general* (9, 11, 13, 18, 19, 21, 22). Prevention of NCDs and chronic illness is also emphasized in these strategies' visions. Further essential keywords of the strategies on NCDs in general include high quality of life (13, 18, 21), healthy lifestyles (18, 21), health-promoting settings/environment (18, 22), and management of chronic conditions (11, 13).

The strategies on *specific* diseases usually also formulate visions or aims that were, however, very heterogeneous. The documents related to mental health, e.g., aim to “improve mental health and wellness” (16, 25), “raise awareness for mental health” (16), “prevent and detect early” (14), and “ensure access to high-quality services/effective treatment” (14, 25). The strategies on chronic respiratory diseases target to “improve the lives [of all Australians] through better lung health” (12) and to “improve services for people with chronic obstructive pulmonary disease and asthma” (24). The aims of the diabetes strategies ranged from developing and implementing an “integrated and coordinated approach for reducing the social, human and economic impact of diabetes” (20), over “improving quality of life and reducing premature death” (15), to “improving outcomes for people living with, or at risk of, diabetes” (17). Strategies on CVDs focus on “improving health and social care outcomes across the population” (23) and on “ensuring an integrated and quality-assured approach in the management of cardiovascular diseases” (26).

**Figure 1:**
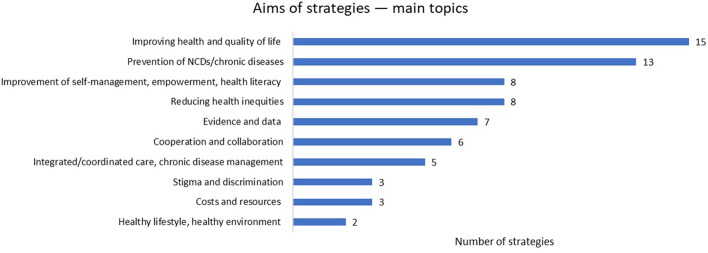
This figure presents the aims of strategies and their main topics.

Most strategies also provide more specific objectives or targets. Various topics addressed in the strategies' aims or objectives are summarized in (Table 3 and Figure 1).

**Table 3:** Topics addressed in the aims/objectives of included strategies.

**Topic**	**Specification (examples)**	**Number of strategies [ref]**
Improving health in general/ quality of life	living healthier lives, stay healthy, promote health, improve quality of life, children grow up healthier, improve health outcomes across the population, improve respiratory health and wellbeing of all communities, improve lives through better lung health, achieve best possible mental health, improve mental health and wellbeing of the population, improve outcomes for people living with diabetes, actively change cardiovascular health for the better, mobilize multisectoral and evidence-based action to promote healthy living	15 (9, 11–13, 15–19, 21–26)
Prevention of NCDs/ chronic illness	prevention of NCDs/ chronic illness/ COPD/ lung conditions/ CVDs/ diabetes/ mental illness and suicide/ depression; reducing the increase in the burden of disease caused by NCDs, reducing premature deaths due to NCDs, effective prevention, mobilize multi-sectoral and evidence-based action to prevent chronic disease and injuries, focus on prevention for a healthier Australia, early detection of mental illness/ depression/ diabetes/ CVD/ COPD	13 (9, 11–16, 18–20, 22, 24, 26)
Self-management, empowerment, health literacy	shared decision-making and self-management, encouraging self-management, person-centered approaches, support self-care and self-management (empowerment), ongoing support as they self-manage their condition, improve health literacy	8 (9, 11–13, 17, 18, 20, 24)
Reduction of health inequalities	focusing on disadvantaged groups (e.g., target priority populations, focus on groups with greatest health risks to significantly reduce discrepancy in [healthy] life expectancy, minimize inequalities between communities, focus on disadvantaged groups and areas with high prevalence), improving equity in access to services (e.g., high-quality health care irrespective of background or personal circumstances, equitable access, equity in access to health promotion and prevention)	8 (11–13, 16, 18, 20, 22, 24)
Evidence, data	evidence-based action, evidence-based services, relevant and current evidence informs best practice; improve data base, increase research capacity, strengthen prevention and care through research, evidence and data	7 (11–13, 15, 17, 19, 20)
Cooperation, collaboration	collaboration and partnerships, collaboration and cooperation, multisectoral action, foster/ reinforce coordination and collaboration at all levels, network stakeholders, coordination and integration of care across services, settings, technology and sectors	6 (13, 15, 16, 19–21)
Integrated care, coordinated services, management of chronic conditions	integrated and patient-centered care, model of care for integrated prevention and management of chronic diseases, coordinated care across the health sector, integrated management, integrated and coordinated approach, proactive approach to early identification, diagnosis and intervention	5 (11, 15, 20, 21, 26)
Stigma, discrimination	reduce stigma and discrimination, raise awareness, reduce social isolation, assure the rights of people with mental illness and enable them to participate meaningfully in society	3 (12, 14, 25)
Costs, resources	reduce the increase in costs due to NCDs, better use of resources, achieve best value with public resources	3 (13, 17, 18)
Healthy lifestyle, healthy settings	target group-specific communication of importance of healthy lifestyle, empowerment to maintain healthy lifestyle; health-promoting environment, create or improve structures to facilitate a healthy lifestyle	2 (18, 21)

*COPD, chronic obstructive pulmonary disease; CVD, cardiovascular disease; NCDs, non-communicable diseases*.

#### Additional Documents

4 strategies provide additional documents containing further information, e.g., on the implementation process (11, 14, 20) or on specific measures to be carried out within the framework of the strategy (18). 7 strategies provide evaluation reports (9, 16, 18, 21, 24, 25) or detailed documents on relevant outcomes and indicators (18, 20, 25). Some strategies also refer to previous or complementing strategies of the respective countries (16, 17, 19, 26).

### Implementation Process

#### Time Frame

14 of the 18 national strategies provide information on the time frame they were developed for. This period ranges from 2 years to a maximum of 10 years. For 7 strategies (19–22, 24–26), the specified time frame has already expired^2^, whereas 7 of the included strategies (10, 11, 13–15, 17, 18) are still in implementation. 4 strategies do not specify the time frame (9, 12, 16, 23).

#### Involved Stakeholders

All of the included strategies mention a range of different stakeholders involved in the strategy's development and/or implementation. The most frequently named stakeholders include:

- government/ministries (at federal, state, municipal level): 18 strategies (9–26);
- health care providers/professional groups: 16 strategies (9–18, 20, 22–26);
- patients and their families/carers: 9 strategies (11–14, 16, 17, 23–25);
- medical associations/commissions: 7 strategies (12, 15, 18, 21, 23, 24, 26);
- various non-governmental and non-profit organizations: 7 strategies (12, 13, 16, 18–20, 22);
- researcher, academic institutions: 6 strategies (9, 12, 13, 16, 19, 26);
- (public and private) health insurances: 4 strategies (9, 12, 13, 22);
- civil society/the “public”: 4 strategies (16, 21, 23, 26).

#### Organizational and Governance Conditions

The national strategies and action plans are described as, for example, a “continuous process,” an “instrument of dialogue” (21), a “guiding framework” for all stakeholders (18), a “frame that structures the efforts” (19), an “overarching policy” (13), a “vision supported by high-level goals” (20), and a “comprehensive, collaborative and evidence-based approach” (12).

Several countries developed their overarching structures, i.e., so-called Boards, Advisory Groups, or Networks, for the organizational and content-related implementation of the strategies. These include, for example, the English “Mental Health Strategy Ministerial Advisory Group” (25), the “Diabetes Network” of the Northern Ireland Diabetes Strategy (17), the English “Respiratory Programme Board” (24), the administrative office of the German “National Action Plan” (21), the committee of the German “Health Targets” (9), the strategic implementation committee of the Swiss “NCD strategy” (18), or the “Mental Health Commission of Canada” (16). These structures usually bring together the various relevant stakeholders and monitor the strategy's implementation to achieve the formulated objectives and visions. Other countries use already existing structures for coordinating the implementation; e.g., the Finnish Ministry of Social Affairs and Health will implement the proposals of the “Mental Health Strategy” (10). For the development and implementation of specific actions and measures, some strategies report on the commissioning of topic-specific working groups, e.g., (9, 15, 21).

#### Monitoring/Evaluation

All included strategies provide information on their planned processes regarding monitoring and evaluation. 5 strategies indicate that a review will be conducted to assess progress after 3 (13, 20) or 5 years (12, 16, 26). 14 documents mention that relevant indicators and outcomes have already been defined as part of an evaluation concept (9–11, 13–18, 20–23, 25). 3 evaluation reports are already available; these include a process evaluation of the German health targets (9), an interim evaluation of the Swiss NCD strategy (18) and a final evaluation report of the German National Action Plan (21).

#### Funding

The information contained in the strategies on financing is heterogeneous and mostly not detailed. 3 documents do not provide funding information (13, 14, 20). Some strategies only disclose the funding of the strategy development [e.g., the respective ministries (10, 12, 26)], while other documents also provide information on the financing of the specific measures planned within the framework of the strategy. The Swiss NCD strategy presents the most detailed insight into the funding plans for specific measures: Swiss funding sources for NCD prevention projects are Health Promotion Switzerland (consisting of annual contributions from each insured person), the Tobacco Prevention Fund (financed by the levy per pack of cigarettes sold) and the “Alkoholzehntel” (cantons receive 10% of the net revenue from the alcohol tax). Additionally, activities are financed by the regular budgets from the cantons and the federal government (18).

Another example is the Dutch strategy indicating that all involved stakeholders contributed to the “National Prevention Programme” by direct investments from their budgets, without mentioning additional resources (22). The German strategy states an annual budget of 5 million euros to implement the “National Action Plan” for the period 2008–2010 (21). The United Kingdom's strategy on CVDs mentions that it has been developed under the premise that there will not be additional funding, aiming at using resources more efficiently (23).

#### Actions/Activities to Reach the Aims/Objectives of the Strategy

All of the included strategies provide information on specific activities or measures planned to be carried out within the implementation of the strategy to reach the respective aims. These are usually structured according to the objectives, “strategic directions,” “priority areas” or “fields of action”. For further analysis, the listed activities are grouped by 7 topics (see Supplementary Material):

- Health promotion, primary prevention (incl. behavioral and structural prevention): e.g., promote healthy eating and physical activity; strengthen tobacco and alcohol prevention; facilitate healthy choices; promote healthy local environments and settings;
- Self-management, health literacy: e.g., support people to learn more about their chronic condition and its management; improve access to structured self-management programs; strengthen (mental) health literacy;
- Early detection, screening: e.g., promote health checks; develop and disseminate evidence-based practice recommendations for early detection of risk factors;
- Disease management, integrated care: e.g., develop integrated pathways between primary and secondary care; implement concepts for patient-centered, coordinated care; ensure effective transfer, discharge and referral pathways between health care services;
- Target group-specific measures, populations at high risk: e.g., integrate target-group oriented measures for each age group; address the specific needs of vulnerable groups; ensure equity of access for all groups;
- Activities outside the health care sector: e.g., promote further implementation of tobacco control measures; further develop cooperation with the economy and facilitate healthy choices; target multiple settings (such as schools, workplaces, communities);
- Digital technologies: e.g., adapt offers in the areas of patient education, self-management and self-help, taking into account modern technologies; support access to telemedicine consultations.

## Discussion

This article aimed to provide an overview of national strategies for preventing and managing NCDs in selected countries. We included 4 NCDs, which are CVDs, chronic respiratory diseases, diabetes type II and depression. We searched for national strategies in various databases and selected 8 countries and their strategies (*n* = 18). Main characteristics and implementation process information from the 18 included strategies were extracted.

Most of the strategies provide information on prevention *and* disease management and formulate rather broad overarching aims or visions (e.g., “stay healthy” or “living healthier lives”) as well as more specific targets that differ across strategies. The strategies were developed for short to intermediate time frames from 2 to a maximum of 10 years. A range of different stakeholders are involved in the development and/or implementation of the strategies, e.g., ministries, health care providers, patients and the civil society, health insurances, as well as research institutions. All analyzed strategies include information on their planned monitoring and evaluation processes. Information on funding is heterogeneous and usually does not go into detail. All of the included strategies provide information on the measures and activities planned to be carried out within the implementation of the strategy, usually structured according to the respective targets, strategic directions or fields of action.

The analysis of the national strategies showed that the degree of organization of the identified strategies and policies differed considerably between the countries. The most elaborated and structured strategy is the Swiss NCD strategy. As part of the Health2020 strategy^3^, the “National Strategy for the Prevention of Non-communicable Diseases 2017–2024” (18) was presented in 2016. The NCD strategy includes actions in 3 main areas: population-related health promotion and prevention, prevention in health care, and prevention in the economy and the world of work. All measures to be carried out within the implementation of the strategy can be found in the associated action plan (27). Regarding monitoring and evaluation, Switzerland also provides detailed information on the indicators to be used as the basis for an NCD monitoring system (28) and has already published an interim evaluation report (29) as well as annual reports.

Other countries, however, do not provide such detailed information on concrete activities and the implementation process. Some strategies remain rather superficial without mentioning specific measures to be carried out to reach the strategy's aims. For some countries, we could not identify any NCD strategies at all. For example, this was the case for Sweden. Sweden does not have an overall strategy for NCDs, but the work with alcohol, tobacco, eating habits and physical activity is instead included in various governing documents.^4^ A comparison across the EU Member States shows that Sweden has the highest healthy life years at birth, with 74 years for men (30). This demonstrates that there may be a number of different successful approaches for tackling NCDs on a strategic level and not a one-size fits all method.

In 2013, the ‘*WHO Global Action Plan for the Prevention and Control of NCDs 2013–2020*' was endorsed by the World Health Assembly. This plan provides the WHO, international partners and Member States with a menu and road map of policy options, including 9 global NCD targets (4). Next to global strategies, regional strategies, such as the “Action Plan for the Prevention and Control of NCDs in the WHO European Region 2016–2025” (3), and national and sub-national strategies exist. However, a report of the UN High-level Meeting on the Prevention and Control of NCDs in 2018 stated that many countries are lagging behind these aims, although lots of proven interventions for NCDs exist (31). In the article of Briggs et al., only one-third of the included 44 policies were explicitly aligned with the WHO global action plan (7). This is in line with our findings: The majority of the included strategies in this paper provide no information concerning the alignment with the WHO global action plan; nearly one-third of this article's strategies were explicitly linked. This is despite the fact that all WHO Member States have decided on the WHO global action plan. While national strategies must naturally reflect local situations, one would still expect to see that the national strategies would follow the WHO strategies and action plan to a certain extent. However, there are other action plans commissioned by the WHO that may be relevant for disease-specific strategies, such as the WHO Mental Health Action Plan 2013–2020 (32), which was mentioned by the Australian strategy on mental health (14).

The 3 key themes to describe the general aims of NCD strategies identified in the study by Briggs et al. (7), analyzing NCD policies among OECD member states, were better population health, improved service delivery and system-strengthening approaches (such as implementing models of care). Our results also reflected these findings regarding the topics of strategies' aims, e.g., “improving health in general”, or “strengthening coordinated services and integrated care.” However, we identified some further themes such as improvement of self-management, empowerment and health literacy, and reducing health inequalities.

The included strategies address NCD prevention and management from a cross-sectoral “Health in All Policies” approach that goes beyond measures within the health care system. Examples are structural prevention activities concerning nutrition, creating physical activity-friendly urban areas or increasing (mental) health literacy in education and the workplace. The Swiss NCD strategy, for example, explicitly refers to the Model of Health Determinants by (33), including socio-economic, cultural and environmental conditions, living and working conditions (such as housing, work environment, unemployment, education), social and community networks, or individual lifestyle factors (18).

11 of the 18 strategies explicitly stated that patients and/or “the public” were involved in developing the strategy as stakeholders. For developing own strategies, more detailed information on the methods of patient involvement will be necessary in order to guarantee representativeness and well-designed involvement processes. Furthermore, it is noticeable that out of 18 strategies, only a minority mentioned academic institutions, civil society or commercial players as stakeholders, although the involvement of as many stakeholders as possible is crucial for comprehensive national action and successful NCD prevention.

Our analysis of the strategies focussed on adults; however, especially in preventing NCDs, a life course approach is helpful to consider all age groups' needs and address NCD prevention and control in its earliest stages (34). Additionally, a life course approach is crucial to tackling inequalities of health, i.e., the unequal conditions in which people are born, grow, live, work, and age, as well as the inequities in power, money, and resources (35). Importantly, health promotion and prevention in early childhood (especially regarding mental health) have been shown to be particularly promising regarding return on investment (36). An alternative to the involvement of children and adolescents in general NCD strategies would be to develop specific strategic plans for their wellbeing as already implemented in some countries.

We included strategies dealing with one or more of 4 NCDs – CVDs, chronic respiratory diseases, diabetes and depression. Other high-burden NCDs, such as cancer or musculoskeletal conditions, were not the focus of this paper, and we, therefore, cannot provide information on those disease areas. The Austrian Ministry was interested in strategies of other countries to develop, improve or adapt Austrian strategies. Cancer, for example, although the second cause of mortality, was not considered because Austria already applies a comprehensive National Cancer Strategy [Krebsrahmenprogramm Österreich].

Furthermore, some countries (e.g., Sweden) do not have specific NCD prevention strategies but instead have legislation, strategies or policies on risk factors such as tobacco and alcohol control, dietary and physical activity policies, or obesity prevention. These components of NCD prevention are not specifically included in the present analyses, which are therefore limited in giving a full overview of all country activities.

We searched for national NCD strategies and policies using a structured and extensive hand search. We did not perform a systematic literature search in databases because national strategies are often published on websites, e.g., of the respective Ministry of Health. As we did an online search for national strategies, we may have missed strategies not published online. Furthermore, the focus of this article was on governmental strategies and, therefore, other strategies (e.g., issued by non-governmental organizations) are not included.

Another limitation is that some countries only provide strategies in their national language. As we could only include documents in English or German, we had to exclude strategies that were not available in one of these languages. However, we included a broad range of countries with different public health traditions and health systems, focusing on Europe but also involving 2 non-European countries.

We did not address all types of characteristics in the strategies. Regarding the objectives and aims of the included strategies, further interesting aspects would be the time horizon (i.e., short-term, intermediate, long-term) and the quality of these aims, i.e., if they can be considered as SMART (specific, measurable, achievable, realistic, time-bound) (37). These may additionally be considered when developing a new strategy.

It is well-known that the implementation gap is usually an issue, and the actual implementation of national strategies matters for national NCD prevention. Fortunately, the implementation of WHO-recommended NCD policies is increasing over time. However, on average, only less than half of the NCD policies recommended by WHO were implemented of the 151 evaluated countries in 2017 (38).

Despite the aforementioned limitations, the results provide a valid overview of the heterogeneity but also common themes across national strategies of different countries in preventing and managing NCDs.

## Actionable Recommendations

We can derive some actionable recommendations for the development and implementation of national strategies: Firstly, strategies may consider a life-cycle approach and particularly include aims addressing prevention of NCDs in children and adolescents because of the long-term preventive potential.

Many important factors influencing the population's health are beyond individual biological factors (age, gender, genetic factors) and lie outside the health care system, such as in the area of social, educational, environmental, labor market, transport or economic policy. Those should be addressed in the strategies by a “Health in All Policies” approach (39). Not addressing those determinants and the structural prevention approaches entails the risk of reinforcing health inequalities.

Finally, a structured approach of strategies from a macro to a micro level seems crucial to achieving a comprehensive, coordinated overall policy. Strategies need to be regularly evaluated using appropriate methods to measure target achievement. Additionally, implementation parameters need to be sufficiently addressed, and evaluation designs need to be developed before strategies' implementation.

To conclude, many Western countries apply national strategies and policies dealing with NCDs' prevention and/or management. These differ substantially in terms of their level of detail, structure and implementation. The strategies mainly aim at preventing NCDs, improving health and quality of life, strengthening self-management and health literacy, reducing health inequalities, and providing integrated care and coordinated services for chronic conditions. All strategies include information on specific actions and activities to be carried out to reach the aims, but only some strategies give detailed information on these programs and interventions, including their evaluation.

## Author Contributions

LG and IR searched for and selected the strategies and extracted, analysed, and interpreted the data. LG wrote the first draft, which all authors revised critically for important intellectual content. All authors contributed to the conception and design of the work, read, and approved the final manuscript.

## Funding

This study was supported by the Publication Fund of the University of Vienna.

## Conflict of Interest

The authors declare that the research was conducted in the absence of any commercial or financial relationships that could be construed as a potential conflict of interest.

## Publisher's Note

All claims expressed in this article are solely those of the authors and do not necessarily represent those of their affiliated organizations, or those of the publisher, the editors and the reviewers. Any product that may be evaluated in this article, or claim that may be made by its manufacturer, is not guaranteed or endorsed by the publisher.

## Footnotes

^1^No specific publication year can be provided for the German health targets, as the process of formulating health targets has been ongoing since 2000 (9).

^2^The Dutch Strategy is titled as Prevention Programme 2014–2016, but formulates long-term objectives which are to be met by 2030 (22).

^3^The Health2020 strategy has since been superseded by the Health2030 strategy: Available online at: https://www.bag.admin.ch/bag/de/home/strategie-und-politik/gesundheit-2030.html (accessed September 27, 2021).

^4^Anna Jansson (Head of Unit for Health-promoting Lifestyles, Public Health Agency of Sweden), personal communication, 20/05/2021 and 24/11/2021.
