# Supplementary material for: National Strategies for Preventing and Managing Non-communicable Diseases in Selected Countries
Source: Front Public Health. 2022 Feb 10;10:838051. doi: 10.3389/fpubh.2022.838051 (PMC8867176; doi:10.3389/fpubh.2022.838051)
Supplement: Supplementary file 1 [file Table_1.DOCX]

Table A-1: Planned activities within the national strategies by topic

| Topic | Specification (examples) | Number of strategies [ref] |
| --- | --- | --- |
| **Health promotion, primary prevention  (incl. behavioural and structural prevention)** | *Chronic disease/NCDs in general*:  promote healthy eating/ balanced nutrition and physical activity, strengthen tobacco and alcohol prevention; make a population-wide and lifestyle-oriented prevention campaign for a healthy lifestyle; encourage healthy behaviour, facilitate healthy choices; facilitate physical activity in everyday life; promote healthy local environments and settings, strengthen prevention in health care; build chronic disease prevention strategies into clinical pathways | 7  [[9](#_ENREF_9), [11](#_ENREF_11), [13](#_ENREF_13), [18](#_ENREF_18), [19](#_ENREF_19), [21](#_ENREF_21), [22](#_ENREF_22)] |
|  | *Cardiovascular diseases*:  focus on population-oriented prevention and health promotion programs and education measures; combine population-based and high-risk approaches; prioritize actions that promote cardiovascular health (e.g., maintaining a healthy bodyweight, healthy eating, physical activity, reducing salt intake, quitting smoking, responsible alcohol consumption) | 3  [[15](#_ENREF_15), [23](#_ENREF_23), [26](#_ENREF_26)] |
|  | *Chronic respiratory diseases*:  develop prevention strategies for respiratory diseases; support accelerated efforts in reducing smoking prevalence and working towards a tobacco-free society | 2  [[12](#_ENREF_12), [24](#_ENREF_24)] |
|  | *Diabetes*:  focus on population-oriented prevention and health promotion programs and education measures; embed physical activity and healthy eating in everyday life; support people in making healthy choices; establish an approach to the prevention of type II diabetes | 3  [[15](#_ENREF_15), [17](#_ENREF_17), [20](#_ENREF_20)] |
|  | *Depression*:  develop integrated approaches to suicide prevention; improve the physical health of people living with mental illness and reduce early mortality; reduce social and other determinants of mental ill health across all ages; utilize evidence-based approaches in promotion and preventative work within social and health care services | 4  [[10](#_ENREF_10), [14](#_ENREF_14), [16](#_ENREF_16), [25](#_ENREF_25)] |
| **Self-management,**  **health literacy** | *Chronic disease/NCDs in general*:  targeted health messages and education; enable people to lead a healthy lifestyle and strengthen self-responsible behaviour; support people to learn more about their chronic condition and its management; give generally understandable information about the clinical picture and treatment options; strengthen self-management of people with chronic conditions, implement ‘self-management support’ framework locally | 5  [[9](#_ENREF_9), [11](#_ENREF_11), [13](#_ENREF_13), [18](#_ENREF_18), [21](#_ENREF_21)] |
|  | *Cardiovascular diseases*:  ensure access to education to support self-management; strengthen health literacy; adapt offers in the areas of patient education/ self-management/ self-help by taking into account modern technologies; increase awareness by the general public of cardiovascular risk factors and levels of risk associated with them through undertaking media and education campaigns | 3  [[15](#_ENREF_15), [23](#_ENREF_23), [26](#_ENREF_26)] |
|  | *Chronic respiratory diseases*:  deliver awareness and education campaigns to increase knowledge of good lung health and symptoms of lung conditions; provide tools, information and support services for patients to support effective self-management practices; develop and pilot innovative technologies and strategies that support patients to be actively involved in their lung health | 2  [[12](#_ENREF_12), [24](#_ENREF_24)] |
|  | *Diabetes*:  enhance access to structured self-management education programs; agree a menu of quality assured Structured Diabetes Education programs; strengthen health literacy; adapt offers in the areas of patient education/ self-management/ self-help by taking into account modern technologies | 3  [[15](#_ENREF_15), [17](#_ENREF_17), [20](#_ENREF_20)] |
|  | *Depression*:  increase mental health literacy and skills in early childhood education, in the workplace and services for older adults; offer people age and developmentally appropriate information; reduce stigma and discrimination by building awareness and knowledge about the impact | 2  [[10](#_ENREF_10), [25](#_ENREF_25)] |
| **Early detection,**  **screening** | *Chronic disease/NCDs in general*:  early detection of risk factors; promotion of health checks, integrated risk assessments and evidence-based screening programs | 4  [[9](#_ENREF_9), [11](#_ENREF_11), [13](#_ENREF_13), [22](#_ENREF_22)] |
|  | *Cardiovascular diseases*:  develop, adopt and disseminate evidence-based, nation-wide practice recommendations for early detection of key risk factors, support the successful implementation of the NHS Health Check Program; develop new tools to support case finding in primary care; develop protocols for risk assessment and early detection of specific CVDs | 3  [[15](#_ENREF_15), [23](#_ENREF_23), [26](#_ENREF_26)] |
|  | *Chronic respiratory diseases*:  enhance early accurate diagnosis and assessment of severity to ensure late diagnosis is minimized | 2  [[12](#_ENREF_12), [24](#_ENREF_24)] |
|  | *Diabetes*:  increase recognition and awareness of type II diabetes and early detection among health care providers and the community; promote increased use of risk screening tools and early management of diabetes with a focus on high-risk groups; develop, adapt and disseminate evidence-based, nation-wide practice recommendations for early detection of key risk factors | 3  [[15](#_ENREF_15), [17](#_ENREF_17), [20](#_ENREF_20)] |
|  | *Depression*:  identify mental health problems and intervene early across all age groups | 1  [[25](#_ENREF_25)] |
| **Disease management, integrated care** | *Chronic disease/NCDs in general*:  develop integrated pathways between primary and secondary care; develop patient-centred care planning approach; develop Chronic Disease Specialist Teams; offer evidence-based targeted interventions for at-risk people and populations; provide efficient, effective and appropriate care to support people with chronic conditions to optimize quality of life; ensure effective transfer, discharge and referral pathways between healthcare services | 2  [[11](#_ENREF_11), [13](#_ENREF_13)] |
|  | *Cardiovascular diseases*:  implement concepts for patient-centred, coordinated care; promote regional networking of existing services in the areas of cardiovascular prevention and rehabilitation, identify how to incentivize and support primary care consistently to provide good management of people with or at risk of CVD, improve acute care; develop structured clinical care; prioritize effective management of hypertension in primary care | 3  [[15](#_ENREF_15), [23](#_ENREF_23), [26](#_ENREF_26)] |
|  | *Chronic respiratory diseases*:  provide chronic disease management and proactive management of all disease severities and any co-morbid conditions and responsive episodic care around the needs of the patient; revise, disseminate and implement evidence-based clinical practice guidelines and tools for lung conditions; investigate and promote equitable access to evidence-based diagnostic tests, medicines and novel treatments | 2  [[12](#_ENREF_12), [24](#_ENREF_24)] |
|  | *Diabetes*:  implement concepts for patient-centred, coordinated care; promote the Shared Decision Making approach; take into account the specific needs of multimorbid patients; improve the experience of care in hospital for people living with diabetes but admitted for other reasons; reduce the occurrence of diabetes-related complications; develop nationally agreed guidelines, local care pathways and complications prevention programs, provide high-quality hospital care | 3  [[15](#_ENREF_15), [17](#_ENREF_17), [20](#_ENREF_20)] |
|  | *Depression*:  support integrated planning and service delivery at the regional level; develop, implement and monitor national guidelines to improve coordination of treatment and supports; make safety and quality central to mental health service delivery; provide/improve access to the right combination of services, treatments and supports, when and where people need them; ensure high-quality care and treatment in the least restrictive environment, in all settings; ensure appropriate, effective transition between services when necessary, without discriminatory, professional, organization or location barriers getting in the way; ensure somatic healthcare for people with mental and substance abuse disorders | 4  [[10](#_ENREF_10), [14](#_ENREF_14), [16](#_ENREF_16), [25](#_ENREF_25)] |
| **Target group-specific measures,**  **populations at high risk** | *Chronic disease/NCDs in general*:  integrate target-group-oriented measures for each age group; specifically, address children and young people as well as adults and older people, target priority populations, e.g., deliver services in a culturally safe way involving people from the same cultural background | 4  [[9](#_ENREF_9), [13](#_ENREF_13), [18](#_ENREF_18), [21](#_ENREF_21)] |
|  | *Cardiovascular diseases*:  promote equity by addressing the specific needs of vulnerable groups | 1  [[15](#_ENREF_15)] |
|  | *Chronic respiratory diseases*: - | - |
|  | *Diabetes*:  promote equity by addressing the specific needs of vulnerable groups; conduct formal needs assessments for particularly vulnerable people to inform future service models and improve outcomes; reduce the impact of diabetes among pregnant women with pre-existing or gestational diabetes, among Aborigines and Torres Strait Islander peoples and other priority groups (culturally and linguistically diverse people, older Australians, Australians living in rural and remote areas) | 3  [[15](#_ENREF_15), [17](#_ENREF_17), [20](#_ENREF_20)] |
|  | *Depression*:  ensure equity of access for all groups, including the most disadvantaged and excluded to high-quality, appropriate, comprehensive services; reduce disparities in risk factors and access to mental health services, and strengthen the response to the needs of diverse communities; improve mental health services and supports by and for immigrants, refugees, ethno-cultural and racialized groups; improve Aboriginal and Torres Strait Islander mental health and suicide prevention; work with First Nations, Inuit, and Métis to address their distinct mental health needs, acknowledging their unique circumstances, rights, and cultures; develop accessible and versatile services which can be provided in the context of the clients’ everyday surroundings, particularly for people who are difficult to reach, at risk of social exclusion, or groups who are vulnerable due to their cultural or social status; use outreach services to reach those who are particularly difficult to reach | 4  [[10](#_ENREF_10), [14](#_ENREF_14), [16](#_ENREF_16), [25](#_ENREF_25)] |
| **Activities outside the health care sector** | *Chronic disease/NCDs in general*:  improve the framework conditions with regard to the promotion of healthy eating and physical activity within educational and care community facilities; promote further implementation of tobacco control measures concerning tobacco advertising and availability of tobacco products; further develop cooperation with the economy and facilitate healthy choices (e.g., 'Nutri-Score', '5 a day' campaign); improve healthy working (e.g., focus on work-related stress, special attention for employees with a disability or chronic condition); target multiple settings (e.g., schools, workplaces, communities) | 5  [[9](#_ENREF_9), [13](#_ENREF_13), [18](#_ENREF_18), [21](#_ENREF_21), [22](#_ENREF_22)] |
|  | *Cardiovascular diseases*:  promote structural prevention (e.g., promote physical activity-friendly urban areas, reduction of sugar, salt and fat in food, introduce food labelling, create incentives for healthy behaviour) | 1  [[15](#_ENREF_15)] |
|  | *Chronic respiratory diseases*:  deliver awareness and education campaigns to improve knowledge of occupational hazards that affect the lungs and to promote safe workplaces/ ensure employers are doing all they can to protect staff and encourage good lung health | 2  [[12](#_ENREF_12), [24](#_ENREF_24)] |
|  | *Diabetes*:  promote structural prevention (e.g. promote physical activity-friendly urban areas, reduction of sugar, salt and fat in food, introduce food labelling, create incentives for healthy behaviour), embed physical activity and healthy eating in everyday life (e.g., workplaces, schools and communities); increase the availability of and demand for healthier food or reduce the availability of and demand for unhealthy food (e.g., through continued implementation and targeted education on the Front-of-Pack Labelling) | 2  [[15](#_ENREF_15), [20](#_ENREF_20)] |
|  | *Depression*:  increase mental health literacy and skills in early childhood education/ schools, workplace, and services for older adults; implement practical help for families via legislative processes in order to reduce poverty in families, create benefits and support parenting; develop a more family-friendly workplace/ create mentally healthy workplaces; increase the capacity of families, caregivers, schools, post-secondary institutions and community organizations to promote the mental health of infants, children and youth, prevent mental illness and suicide wherever possible, and intervene early when problems first emerge; reduce the over-representation of people living with mental health problems and illnesses in the criminal justice system and provide appropriate services, treatment and supports to those who are in the system; increase access to housing with supports, and to income, employment, and education support for people living with mental health problems and illnesses, and provide greater support to families; build care and support around outcomes that matter to individuals to enable them to live the lives they want to live, including good relationships, purpose, education, housing and employment | 3  [[10](#_ENREF_10), [16](#_ENREF_16), [25](#_ENREF_25)] |
| **Digital technologies** | *Chronic disease/NCDs in general*:  in the Irish 'model of care' (defining 5 levels of services), services on level 0 (‘living well with chronic disease’) include telehealth and remote monitoring; technology broadens access to health services, including appropriate use of telehealth and digital health options | 2  [[11](#_ENREF_11), [13](#_ENREF_13)] |
|  | *Cardiovascular diseases*:  adapt offers in the areas of patient education/ self-management/ self-help taking into account modern technologies; support patients in their daily lives through adequate communication (including modern communication technologies) | 1  [[15](#_ENREF_15)] |
|  | *Chronic respiratory diseases*: - | - |
|  | *Diabetes*:  adapt offers in the areas of patient education/ self-management/ self-help taking into account modern technologies; support patients in their daily lives through adequate communication (including modern communication technologies); support current access to flexible telemedicine consultations (e.g., medical consultation for diabetes, eye screening program, telephone-based lifestyle coaching) and explore the expansion of telehealth services; ensure the availability of telehealth and internet medical services for Australians living in rural and remote areas; explore the role of digital technology and social media in self-management; establish a diabetes care pathway with the electronic care record, and a portal through which people living with diabetes can manage their own health information and interact with clinicians | 3  [[15](#_ENREF_15), [17](#_ENREF_17), [20](#_ENREF_20)] |
|  | *Depression*:  establish a digital information centre for effective mental health promotion and prevention of mental health problems | 1  [[10](#_ENREF_10)] |
